# Neural signatures of response planning occur midway through an incoming question in conversation

**DOI:** 10.1038/srep12881

**Published:** 2015-08-05

**Authors:** Sara Bögels, Lilla Magyari, Stephen C. Levinson

**Affiliations:** 1 Max Planck Institute for Psycholinguistics, Nijmegen, The Netherlands; 2 Pazmany Peter Catholic University, Faculty of Humanities and Social Sciences, Department of General Psychology, Budapest, Hungary; 3 Donders Institute for Brain, Cognition and Behaviour, The Netherlands

## Abstract

A striking puzzle about language use in everyday conversation is that turn-taking latencies are usually very short, whereas planning language production takes much longer. This implies overlap between language comprehension and production processes, but the nature and extent of such overlap has never been studied directly. Combining an interactive quiz paradigm with EEG measurements in an innovative way, we show that production planning processes start as soon as possible, that is, within half a second after the answer to a question can be retrieved (up to several seconds before the end of the question). Localization of ERP data shows early activation even of brain areas related to late stages of production planning (e.g., syllabification). Finally, oscillation results suggest an attention switch from comprehension to production around the same time frame. This perspective from interactive language use throws new light on the performance characteristics that language competence involves.

There is a striking but largely unexplored puzzle at the heart of the language sciences. The core ecological niche for language use is conversational interaction, where speakers take rapid turns at talking. The puzzle is that the changeover between speakers is very quick, but the latencies involved in planning language production are three to ten times longer (depending on the nature of the turn under construction). This implies that to achieve this timing, participants must start production planning already during the incoming turn. Thus, there must be significant overlap between language comprehension processes and production ones – such dual-tasking forms an interesting challenge for the system since both processes draw on largely the same cognitive and neural resources.

Earlier work has established the tight timing of turn-taking in conversational interaction^1^, with a gap of about 200 ms on average (about the duration of a syllable) between turns^2^,^3^. The latencies involved in language production are also well studied: it takes at least 600 ms to plan the production of a single frequent word from conception to the beginning of speech^4^, and 1500 ms to plan the simplest sentence^5^. These facts seem to imply that conversational participants are already planning their response mid-way through the incoming turn. That suggests in turn that listeners can predict the end of turns, for which there is some direct evidence^6^, although how exactly this is done, for example the role of intonation versus syntax, remains unclear^7^,^8^,^9^. But we know little about the underlying cognitive processes involved in such turn-taking because psycholinguistic studies have largely looked at the two processes of comprehension and production separately, divorced from an interactive context. Here we show for the first time, using EEG, that in interactive language use listeners indeed begin planning their response as soon as they grasp the point or purpose of the incoming turn, up to several seconds before the end of that turn.

Given the predictive comprehension and early production implied by turn-taking, a crucial locus of investigation is what cognitive processes are going on in the recipient during an incoming turn to which he or she must respond. Using electroencephalography (EEG) as a non-invasive way of tapping into these processes with an excellent time resolution, we designed an innovative interactive turn-taking paradigm in the form of a quiz game (see Methods). This preserved the essential turn-taking structure, and is a familiar genre of interactive language use. Sitting in a shielded chamber, participants were asked to respond quickly (within 5 seconds) to Dutch quiz questions, and received live feedback from the experimenter. Unbeknownst to the participant the quiz questions themselves were pre-recorded by the same experimenter, providing a high degree of experimental control across participants.

To vary when participants could start planning their answers, we created two different conditions.

1. EARLY: Which character, also called **007**, appears in the famous *movies*?
2. LATE: Which character from the famous *movies*, is also called **007**?

In ‘EARLY’ questions (see example 1) the critical information essential to answer the questions (*007,* in bold) was available early on, whereas in ‘LATE’ questions (see example 2), this information was not available at the equivalent position (*movies,* in italics), but only became available at the very end of the question. The position in the middle of the sentence (onset of the critical word in the EARLY condition) is hereafter referred to as the first time-locking point (or TL1) and the onset of the last word of the sentence is referred to as the second time-locking point (or TL2). Answers constituted one word or a small phrase. We were interested in how soon participants would start planning the production of their answer after the critical information became available, especially in the EARLY condition. Crucially, to distinguish production vs. comprehension processes, a control experiment was conducted, in which participants listened to the same quiz questions but instead of answering them, were asked to remember the questions and were tested using probes.

## Results and Discussion

### Behavioral Analyses

For the main experiment, a linear mixed-effects model with response time as the dependent variable, condition (EARLY, LATE) as the main predictor, and random intercepts for participant and item, showed that responses relative to question offset in the EARLY condition (M = 640 ms, mode around 400 ms) were faster than in the LATE condition (M = 950 ms; mode around 750 ms; intercept: β = 312; LATE: β = 328; t = 17.31, p < .0001; see also Supplementary Fig. 1). This difference in response times between the two conditions suggests that participants did some part of production planning in overlap with comprehension for EARLY questions, but gives no information about when exactly planning began. The EEG data were analyzed to give a more precise answer to this question.

### ERP Analyses

**Figure 1:**
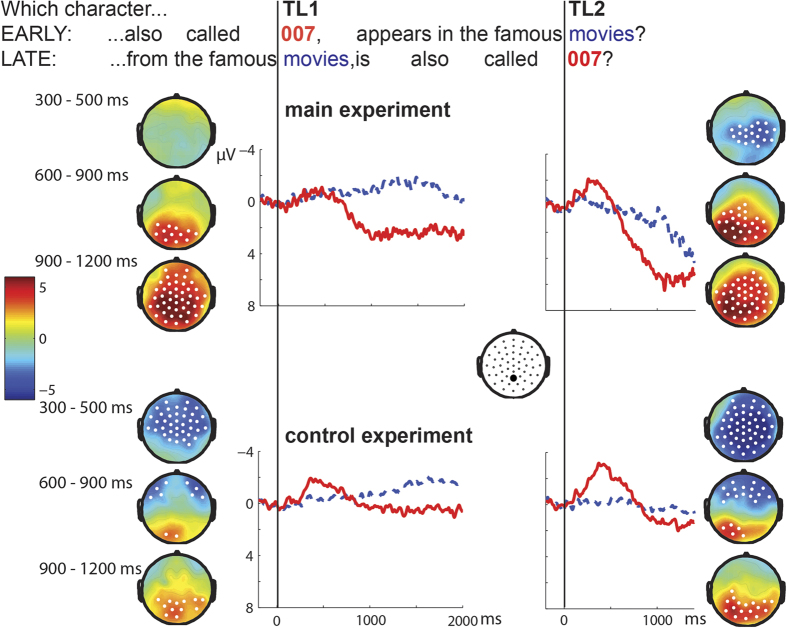
Grand average ERPs for a representative electrode (Pz). Critical words are always indicated by red solid lines and equivalent positions by blue dashed lines. Topographical plots are given for the N400 time window (top: 300–500 ms) and two time-windows for the positivity (middle: 600–900 ms; bottom: 900–1200 ms). Colors indicate T-values. Electrodes that show a significant effect in more than 70% of the time window are highlighted in white.

We computed event-related brain potentials (ERPs) based on the EEG data from 64 electrodes, time-locked to TL1 and TL2. Figure 1 illustrates the contrasting findings between the main and control experiments. In the main experiment, we first found a small negative effect (109–522 ms; p = .01) in TL2 (for LATE vs. EARLY questions) only. This effect was larger and appeared at both time-locking points in the control experiment (TL1, EARLY vs. LATE: 246–980 ms; p = .007; TL2, LATE vs. EARLY: 72–1007 ms; p < .001). We interpret this as an N400 effect^10^ plausibly related to the predictability of the words (critical words were less predictable, at least when they appeared at the end of the question, see Materials in Methods). The larger N400 effect in the control study was backed up by a statistical comparison of the two experiments at TL2, yielding an effect between 360 and 500 ms (p < .05; however, note that this is a between-participants comparison). It could be explained by a stronger focus on comprehension required to remember the complete questions in the control experiment, at a time when participants in the main experiment may have already started focusing on production processing (see also time-frequency analysis below).

Second, and most importantly, we found a large positive effect, starting around 500 ms after the onset of the critical information in both TL1 (EARLY vs. LATE: 564–2000 ms; p < .001) and TL2 (LATE vs EARLY: 516-1283 ms; p < .001). In the control experiment, in which participants did not answer the questions, positivities were present as well (TL1, EARLY vs. LATE: 614–2000 ms; p < .001; TL2, LATE vs. EARLY: 582–1400 ms; p = .006), but they were reduced in size, as confirmed by statistically comparing the two experiments (TL1: 708–1422 ms; TL2: 546–1125 ms; ps < .01). Note that the smaller positivities in the control experiments are not caused by the larger N400 effects found there, as shown by two additional analyses (baselining at the N400 window and a peak-to-peak analysis, see Supplementary Fig. 2). Earlier studies have found this kind of polarity and timing in the EEG signal associated with syntactic violation or mismatch (P600^11^), but this does not seem a plausible interpretation in this context. Since the experiments were designed to differ on the presence or absence of an overt response, we relate the larger positivity in the main experiment to response planning. The smaller positivity in the control experiment is probably related to the difference in task, namely comprehension (and memory) without production (see localization results below). A few earlier ERP studies also looked at (overt or implicit) language production, in the form of picture naming^12^,^13^,^14^,^15^,^16^,^17^,^18^. Most of these did not include a passive condition without production^13^,^14^,^15^,^16^,^17^,^18^. Still, visual inspection of the figures in these studies suggests waveforms going more positive around 300 ms after picture onset for most conditions that involve some kind of (implicit or overt) language production. These positivities in most cases appear to be largest at posterior or parietal electrodes^13^,^15^,^16^,^17^,^18^, as in the present study. Critical comparisons in the studies mostly involved naming conditions with different degrees of difficulty, yielding a larger positivity for more difficult items (e.g., accompanied by more difficult distractor words). Interestingly, one study that did compare overt and covert naming with passive viewing^12^ found a clearly larger centroparietal positivity for both types of naming than for passive viewing of pictures. The later timing of the positivity in the present study (i.e., around 600 ms) is possibly caused by a longer time needed to find an answer to the question than to find a name for a picture.

**Figure 2:**
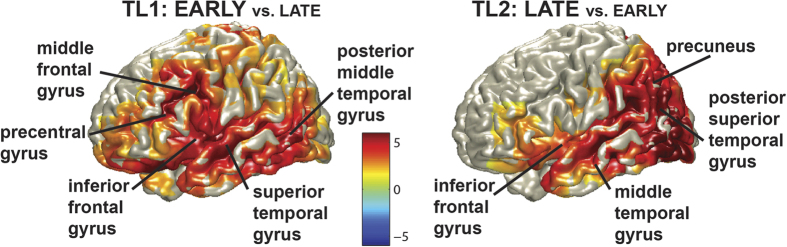
Localizations of the positivities in the ERPs (600–1100 ms) of the main experiment. Localizations for the effects at TL1 (EARLY vs. LATE) are shown on the left and for that at TL2 (LATE vs EARLY) are shown on the right. Only the left hemisphere is shown since activations occurred mainly in this hemisphere. Colors indicate T-values.

A localization of the positivities was performed between 600 and 1100 ms. In the main experiment, positivities for both TL1 (EARLY vs. LATE: one cluster, p < .001) and TL2 (LATE vs. EARLY: one cluster, p < .001) were localized at distributed sources in the brain, mostly in the left hemisphere (Fig. 2). Local maxima for both question conditions were found in the middle and superior temporal gyrus and the inferior frontal gyrus. In addition, the positivity at TL1 (EARLY vs. LATE) showed local maxima in the middle frontal and precentral gyrus and the positivity at TL2 (LATE vs EARLY) showed a local maximum in the precuneus. These areas overlap extensively with the network of language production areas^4^, and have been related to lemma retrieval and selection, phonological code retrieval, and syllabification. In contrast, the positivities in the control experiment (TL1, EARLY vs. LATE: one cluster, p = .034; TL2, LATE vs. EARLY: one cluster, p = .025) were mainly localized to the anterior and posterior cingulate cortices (see Supplementary Fig. 3), which are not specifically associated with language processing, but rather with attention and attentional control^19^. Participants in the control experiment might need such attentional control to keep to the memory task, while the critical words come in, prompting them to ‘automatically’ try to retrieve the answer (as more than 90% of participants reported to have done). Given that the localizations of the (smaller) positivities in the control experiment point to rather different processes, the localizations of the positivities found in the main experiment are consistent with the interpretation that language production planning, right up to the later stages of phonological retrieval and syllabification, started almost immediately after the answer could be retrieved.

### Time-Frequency Analyses

**Figure 3:**
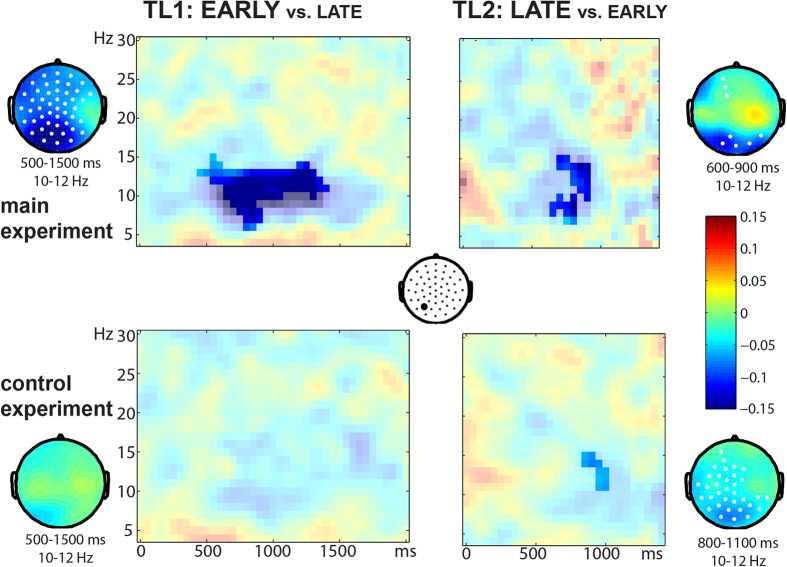
Time-frequency results for a representative electrode (left posterior, see middle). Colors in all plots indicate the relative difference between raw power in the relevant conditions. In the time-frequency plots, the relative difference is given in transparent colors with the statistically significant cluster overlaid in opaque colors. Topographical plots are given for appropriate time windows and for the 10–12 Hz range. Electrodes that are significant in the time window are highlighted in white.

In a further analysis, we computed time-frequency representations of the oscillations of the EARLY and LATE questions, using the same time-locking as for the ERPs. We found that power was modulated around the alpha band (about 9–14 Hz) differentially across the two experiments (Fig. 3). In the main experiment, we found reduced alpha power at TL1 for EARLY vs. LATE questions between about 500–1500 ms after the start of the time-locking point (p < .001). At TL2 we found marginally significant reduced power in the alpha band for LATE versus EARLY questions between about 600 to 900 ms after the onset of the last word (p = .07). To summarize, in the main experiment we found reduced alpha power in both conditions right after the critical information was presented in the questions (e.g., *007*). In contrast, in the control experiment where no overt production was required, no frequency effects were found at TL1. At TL2 marginally significant reduced alpha power was found for LATE vs. EARLY questions between about 800 and 1100 ms after onset of the last word (p = .06). An analysis comparing the two experiments confirmed that the reduced alpha effect at TL1 (EARLY vs. LATE) was stronger in the main experiment than in the control experiment (between about 500 and 1500 ms; p = .004), but we found no differences between the experiments at TL2. Since the reduced alpha power was not present or smaller in the control (comprehension) experiment than in the main (production) experiment, this effect also appears to be related to production preparation.

**Figure 4:**
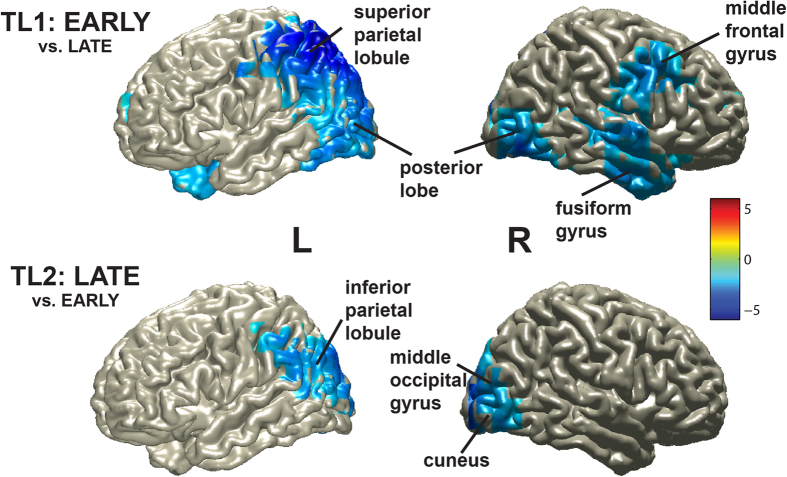
Localizations of reduced alpha power for the critical words in the main experiment. Localizations of the reduced alpha power at TL1 (EARLY vs. LATE) are shown at the top and those at TL2 (LATE vs EARLY) are shown at the bottom. Colors indicate T-values.

These oscillatory effects might be interpreted in two different ways. First, reduced alpha (and beta) power has been associated with motor preparation (often then called mu desynchronization^20^). Thus, this effect could be related to preparing of motor responses needed for producing the answer. Second, alpha power changes have been associated with attentional and working memory effects^21^. To shed more light on the issue, we performed source localizations of the effects in the main experiment. At TL1 (EARLY vs. LATE; 9–14 Hz, 500–1500 ms; one cluster, p = .013) maxima for the reduced alpha effect were localized in parietal/occipital and posterior parts of the brain, most strongly in the left hemisphere (Fig. 4, top). At TL2 (LATE vs. EARLY; 9–14 Hz, 500–1500 ms), we found a marginally significant cluster (p = .08), also localizing to parietal and occipital areas (Fig. 4, bottom). Since these localizations are not in or close to the motor cortex, they therefore do not corroborate an interpretation related to motor preparation. A second interpretation, related to attention, notes that increased alpha power in the posterior (occipital/parietal) part of the brain has been associated with attention to auditory input^21^,^22^,^23^ and a high working memory load^24^, possibly to actively inhibit visual areas that are irrelevant for the task^21^. In the present experiments, participants generally had to attend to the auditory questions and build a mental model of them in working memory, leading to a high level of alpha power in visual cortex. However, at the moment that the answer could be retrieved in the main experiment, attention arguably shifted to preparing production of the answer. This would be at the cost of listening to and comprehending the ongoing question and of keeping a mental model of the question in working memory. This is compatible with a relative decrease of alpha power in parietal/occipital areas at that moment. In addition, retrieval of the correct answer and production preparation might involve visual imagery of the answer (e.g., James Bond), which might also lead to reduced alpha power in visual regions of the brain (cf. earlier findings of reduced alpha/beta power in a word generation task^25^). Such a shift from auditory attention and working memory to other processes (i.e., language production planning) probably does not happen (at least to the same degree) in the control experiment, because participants had to keep their full attention on the incoming speech in order to remember the complete question later on. This interpretation is further corroborated by additional analyses showing that reduced alpha power to the critical word in the main experiment is also found in comparison to a baseline immediately before the critical word (see Supplementary Fig. 4). This is consistent with an account in which participants attend to comprehension until the answer becomes known and then switch to production planning. That such an attention shift might be necessary is corroborated by recent research showing that sustained attention is important for language production processes, especially in a dual task situation^26^. However, this implies that language comprehension might suffer once production planning starts, an interesting avenue for future research.

## Conclusion

We found two EEG correlates of the preparation to respond to questions while they were still incoming—correlates missing or greatly reduced when the task was to memorize the questions rather than respond to them. The first effect was a positivity in the ERPs that appears directly related to language production processes (such as lemma retrieval and selection, phonological code retrieval, and syllabification), as suggested by source localization of the positivity. The second effect was reduced power in the alpha band that we interpret as a switch of attentional resources from comprehension to answer retrieval and production processes. Importantly, both effects started already around 500 ms after the onset of the information that enables participants to retrieve the answer. This indicates that interlocutors started production planning within half a second of hearing the critical information necessary to start answer preparation – in the EARLY questions on average around 2.4 seconds before the end of the question. Additional analyses time-locked to the response (the moment of speaking, see Supplementary Fig. 5) are consistent with this conclusion. These analyses show similar effects as the analyses time-locked to the critical words, but much more spread out in time. This suggests that both the positivity and the alpha reduction are better time-aligned with the onset of the word that enables answer retrieval than with response onset. In other words, most participants appear to start planning soon after the first possible moment that they could start, rather than at a fixed time before they start speaking.

Our conclusions appear to contrast with recent studies^27^,^28^ suggesting that the cognitively demanding aspects of speech planning start close to the end of the previous turn, using a dual-task paradigm. Boiteau *et al.*^27^ used an interactive paradigm whereas in Sjerps and Meyer^28^, participants listened to a computer voice naming one row of pictures and subsequently had to name the other row themselves. One way to reconcile the different studies would be to suggest that early, less cognitively demanding aspects of speech planning occur as soon as possible (present study) whereas later, cognitively demanding aspects occur at the last half second^27^,^28^. However, the present ERP localization results suggest that most of the production planning stages are involved early on. Moreover, if an attention switch from comprehension to production is really necessary, as the present alpha decrease suggests, it seems that speech planning is cognitively demanding from the start. Since it is still early days for investigations of interactive turn-taking, future research is needed to explain the differences in results between the studies. Importantly, Boiteau *et al.*^27^ did not control the position in the turn at which response planning could start, which makes it difficult to draw conclusions about early versus late planning. In terms of ecological validity, in Sjerps and Meyer’s^28^ study the participant’s turn was not semantically contingent on the previous one, as is usually the case in conversation. Furthermore, participants in the Sjerps and Meyer^28^ study saw depictions of what their interlocutor was saying, which might have grabbed their visual attention, leading them to postpone planning the naming of their own pictures.

Assuming that listeners do start planning early, this goes some way to explain how the split-second timing of conversation is achieved: responders begin planning their response as soon as they can, often midway during the incoming utterance. What it leaves unexplained is how interactants also manage to avoid overlapping each other as often as they do. For this, we think an ancillary mechanism must be involved which estimates the timing of the end of the incoming turn, probably by using the syntactic frame and other contextual information to predict the words^6^ and intonational cues to trigger the launch of the response^8^,^9^. Another intriguing question that remains is how comprehension and production processes happen in parallel, especially if they both need overlapping neural circuitry and attentional resources. The present study is among the first to attempt to investigate neural correlates of language processing in an interactive setting, here in the ecologically valid situation of a quiz. We expect that the processes employed by interlocutors in such a context do not differ substantially from those employed in other interactive situations. Future neuroimaging research should venture into even less restricted forms of turn-taking, ultimately free conversation. Such studies can make use of the methods and findings provided by the present study: experimental paradigms using partially pre-recorded and partially live interaction, utterance stimuli that vary in the point at which response can be prepared, and most important of all, the neural signatures that seem related to production planning.

## Methods

### Ethical Approval

All experiments were carried out in accordance with guidelines approved by the *Ethics Committee Faculty Social Sciences* of the Radboud University Nijmegen.

### Participants

For the main experiment, 31 participants were recruited from the participant pool of the Max Planck Institute for Psycholinguistics. Data from 7 participants was excluded from the analysis because of too many artifacts (see EEG data analysis). The 24 remaining participants (8 male, 16 female) had a mean age of 21.3 years old (range 18 to 24). For the control experiment, another 32 participants were recruited from the same pool. Data from 2 participants was excluded from the analysis because of too many artifacts (see EEG data analysis). The 30 remaining participants (8 male, 22 female) had a mean age of 21.1 years old (range 18 to 25). All participants were native speakers of Dutch without hearing impairments. They gave informed consent before participating and received 8 euros per hour for their participation.

### Materials

We used 94 experimental Dutch question pairs in two different conditions. In EARLY questions (see example 1), the ‘answer-recognition’ point (the point at which the answer was clear) occurred early in the question, followed by 8.1 syllables on average (range 5–21) or 4.4 words (range 2–10), whereas in LATE questions (see example 2), this point occurred during the last word (or two words). The questions of each pair differed in the order of the constituents, but furthermore contained the same words as much as possible and fitted with exactly the same answer.

1. EARLY: Welk karakter, ook wel **007**, komt voor in de bekende *films*? “Which character, also called **007**, appears in the famous *movies*?”
2. LATE: Welk karakter uit de bekende *films* heet ook wel **007**? “Which character from the famous *movies* is also called **007**?”

A pretest (20 participants) ensured that the EARLY questions could be reliably answered correctly immediately after the answer recognition point (i.e., critical word offset; M = 91%, range: 50–100%) and the LATE questions could not reliably be answered correctly before hearing the last word (M = 4%, range: 0–35%). The same pretest showed that the average cloze probability of the last word was .07 for the LATE condition and .59 for the EARLY condition (t(93) = 13.89, p < .001). Thirty pairs (EARLY and LATE) of relatively difficult quiz questions served as filler questions, to create a more realistic quiz and to create more of a competition for the prizes (see Procedure). A pretest (10 participants) showed that they were answered correctly 31% of the time on average (range: 10–80%).

All experimental and filler questions were recorded twice by a research assistant (RA) in the EEG room under the same circumstances as during the EEG experiment, and the best token of each question was chosen for the experiment. The experimental quiz situation was simulated by having a participant present who answered the questions. Some of the filler questions (but none of the experimental questions) were recorded with a hesitation or error to create the illusion of live speech. Recorded items were cut around 500 ms before question onset and exactly at question offset using Praat^29^. Five hundred ms of recording noise was then added at the start (fade-in) and 500 ms at the end (fade-out) to make the transition to live speech as smooth as possible.

### Design

The experimental questions could differ on the factor Answer (EARLY, LATE). Two lists were created, which were both administered to half of the participants. List 1 contained half of the experimental and filler questions in the EARLY condition and the other half in the LATE condition, which was reversed for list 2. All items were presented in the same order for all participants. The items were divided into 4 blocks of 31 questions each with pauses in between. Question topics were spread over the 4 blocks and each block contained 7 or 8 difficult filler questions, but never twice in a row. The exact same design was used for the main and the control experiment.

### Procedure

#### Main Experiment

After EEG preparation, participants sat down in a sound proof booth in front of a computer screen. The same RA that recorded the materials instructed them from outside of the soundproof booth via a sound system. The RA used a microphone and participants heard her via loudspeakers. Participants were first informed that the two participants who answered most questions correctly would win a prize. Then, the RA instructed participants to answer quiz questions that varied from easy to difficult. They were urged to focus on giving the right answer, within a deadline of 5 seconds. They should look at a fixation cross on the screen (without moving or blinking) that was presented from between 1500 and 2000 ms before the question started until a button-press by the RA after the answer was given. Afterwards, the RA gave feedback while the word ‘knipperen’ (blink) was visible on the screen, indicating that participants could blink their eyes. They were told that the complete interaction was live, while in reality the questions were played by a button-press from the RA, while the feedback was given live. Participants started with a practice block of 16 questions, followed by the 4 experimental blocks. After the experiment, participants filled out a short questionnaire and received a debriefing sheet, informing them that the questions had been recorded.

In the questionnaire after the experiment, in a first open question, none of the participants reported to have noticed anything strange during the experiment. When asked directly whether they thought (in retrospect) that any of the stimuli they heard could have been recorded, 6 participants (25%) reported that some of the questions could have been recorded. Also 3 other participants (12.5%) reported that the feedback after the questions could have been recorded (although this part was actually live). To keep enough power, we kept all these participants in the analyses. However, we performed additional ERP and time-frequency analyses for the main experiment without the 6 participants that reported that some of the questions might be recorded, to exclude the possibility that these participants could explain some of the differences we found (see Supplementary Fig. 6).

#### Control Experiment

The procedure was the same as in the main experiment except for the following. Participants were instructed that this was a control experiment for an earlier quiz experiment in which participants answered the questions. They were told that the RA read the questions to them live to keep as close to the quiz experiment as possible. Participants were asked not to answer but try to remember the questions. After each block of questions, they saw 10 probe questions on their screen, one by one, and had to indicate for each question whether they had heard it in the previous block or not. Participants received two practice blocks of 20 questions each, to give them an indication of the type of foils they could expect. The foils were very similar to the questions they had heard, differing either in answer or in content. For example, for the quiz question “Which element in coffee is the effective element?”, the foil was: “Which element in coffee wakes you up?”

On average 92% of probe questions were remembered or discarded correctly, showing that participants paid attention to the questions. The questions from both conditions were remembered equally well (T < 1). In the questionnaire after the experiment, in a first open question, none of the participants reported to have noticed anything strange during the experiment. When asked directly, only 3 participants reported that some of the questions might have been recorded.

### Apparatus

EEG was recorded from 61 active Ag/AgCI electrodes using an actiCap^6^. Of these, 59 electrodes were mounted in the cap with equidistant electrode montage referenced to the left mastoid. Two separate electrodes were placed at the left and the right mastoid. Blinks were monitored through a separate electrode placed below the left eye and one of the 59 electrodes in the cap. Horizontal eye movements were monitored through two separate electrodes placed at each outer canthus. The ground electrode was placed on the forehead. Electrode impedance was kept below 10 kΩ. EEG and EOG recordings were amplified through BrainAmp DC amplifiers. EEG signals were filtered online with a band-pass filter between 0.016 and 100 Hz. The recording was digitized online with a sampling frequency of 500 Hz and stored for offline analysis.

### Data Analysis

For behavioral data analysis of the quiz questions, we looked at the numbers of errors in the two conditions and we measured the response time from the end of the question to the start of the answer. Incorrect responses (10%, no difference between EARLY and LATE conditions, p > .8) and responses starting with hesitations (5%) were discarded before the analysis. We ran mixed-effects models on these two variables to assess the effect of Condition (EARLY, LATE) in R.

Preprocessing and statistical analysis of EEG data was conducted using Fieldtrip^30^. Items with incorrect answers were discarded before EEG analysis. For quiz questions, epochs were extracted from 500 ms before the start of a question until 100 ms before speech onset (in the main experiment) to avoid speech artifacts. Only for purposes of artifact rejection, these epochs were filtered with a low pass filter of 35 Hz, detrended, and baselined with a baseline of 200 ms immediately before sentence onset. Epochs containing eye artifacts or other artifacts that exceeded +/− 100 μV (visual inspection) were discarded. Seven participants from the main experiment and 2 participants from the control experiment with less than 18 remaining trials in any of the two conditions were not analyzed further. For the remaining participants in the main experiment an average of 33 trials (range: 18–42) remained for both the EARLY and LATE condition (no significant difference between the conditions; t(23) = .04, p = .97) and for the remaining participants in the control experiment an average of 39 trials (range: 22–47) remained for both the EARLY and LATE condition (no significant difference between the conditions; t(29) = .26, p = .80). Following artifact rejection, smaller epochs were extracted from the questions for analysis. The first time-locking point (TL1) was the onset of the word that constituted the answer recognition point in the EARLY condition (e.g., *007* in example 1). As an equivalent point in the LATE condition, we took the word onset that was closest in time to the same point in the EARLY condition (as measured from sentence onset) for each item (e.g., *movies* in example 2). The second time-locking point (TL2) was the onset of the last word in the LATE condition (also the answer recognition point, e.g., *007* in example 2) and in the EARLY condition (e.g., *movies* in example 1). See Table 1 for temporal measurements regarding the time-locking points. To ensure natural sounding quiz questions, we did not match the critical and equivalent words. This is relatively unproblematic, since our critical comparison was between experiments. In these analyses, the exact same comparison between critical and equivalent words was made for both experiments, so the difference cannot be due to differences between the critical and equivalent words. Furthermore, the majority of equivalent words (and all critical words) were content words, that is, 76% of equivalent words at TL1 and 91% of equivalent words at TL2. To exclude that the relatively few equivalent function words could explain some of the differences we found, we performed additional ERP and time-frequency analyses for both experiments excluding items for which the equivalent words were function words (see Supplementary Fig. 7).

Epochs for analysis consisted of 200 ms before until maximally 2000 ms after TL1 and 200 before until maximally 1400 ms after TL2 since speech started earlier for TL2 (trials were always only included up to 100 ms before response onset). These preprocessed data entered the event-related potential (ERP) and time-frequency (TF; power) analyses. For ERPs, epochs were filtered with a low-pass filter of 35 Hz and baselined with a baseline window of 200 ms immediately before the time-locking point. Then, trials of the same condition were averaged per participant. For time-frequency representations, no filtering or baselining was performed, but a linear trend was removed from the data before the analysis. The power of each frequency between 4 and 30 Hz (with steps of 1 Hz) was calculated on the extracted epochs of individual trials using a Hanning taper^31^ with a window of 500 ms for each frequency. For illustration purposes, relative differences were calculated between conditions, dividing the absolute power difference between conditions by the sum of the power in both conditions (see Fig. 3).

To test for statistically significant differences between conditions and reduce the multiple-comparison problem, we used the cluster-based approach^32^ implemented in the Fieldtrip toolbox for the ERP as well for the TF analysis. This robust method reduces the multiple-comparisons problem and controls family-wise error across participants in time and space. To examine differences between experimental conditions, paired T-tests are performed for each time-point, channel, and frequency (for time-frequency analyses) with a threshold of .05. All time, channel (and frequency) points below the threshold are selected and clustered. Clusters in time, space, and frequency are identified on the basis of proximity of the points (neighbours) in all dimensions of the cluster. Cluster statistics are calculated by taking the sum of t-values in every cluster. To obtain a p-value for each cluster, a Monte Carlo method is used to estimate the permutation distribution of the largest cluster statistic. The permutation distribution is created by 1000 random permutations of the samples of the two conditions. At each randomization, clusters are identified and the largest sum of t-values of the clusters enters the permutation distribution. The proportion of maximum cluster statistics of the permutation distribution that is larger than the observed one is the p-value. The threshold was fixed to p = .05. Analyses were performed within a time-range of 0–2000 ms for the first time-locking point and 0–1400 ms for the second time-locking point, and (for TF analyses) a frequency range of 4–30 Hz. To compare between experiments, we calculated the mean difference between the two conditions for each participant in the two experiments. Following, we used the cluster-based approach described above on these difference scores with the between-participant factor Experiment. To test whether the larger positivities in the main experiment relative to the control experiment could be due to differences in the size of the earlier N400, we performed two additional analyses on the ERPs comparing the two experiments. In the first analysis, a baseline window of 300–500 ms was used (the standard N400 window^33^), neutralizing any differences caused by differences in the size of the N400, and the procedure described above was applied to this data. In the second analysis, for each average per participant, we calculated the minimum value in the 350–450 ms window and the maximum value in the 950–1050 ms window. These two values were subtracted, yielding the peak-to-peak difference between the N400 and following positivity. These values were subjected to the same procedure comparing the two experiments. The results of these analyses are reported in the Results and Discussion section and Supplementary Fig. 2.

To identify sources underlying the electrode-level effects, a BEM (boundary element headmodel)^34^ was used based on a template MRI aligned with the EEG electrode array. Time windows and frequency ranges (in case of time-frequency sources) for the source analysis were chosen based on significant electrode-level effects (600–1100 ms for ERP effects and 500-1500 ms and 9–14 Hz for TF effects). ERP sources were identified using a Linearly Constrained Minimum Variance (LCMV) beamformer^35^, where we calculated a common LCMV filter for the two conditions together per participant. This common filter was then used to transform the participants’ ERP signals into source (voxel) space for comparisons between conditions. For identifying generators of oscillations we employed Dynamic Imaging of Coherent Sources (DICS) beamformers^36^ and also used common filters. Power values were calculated on an equidistant template 3-D grid with a 1 cm resolution. Otherwise no anatomical constraints were imposed on the source localization. A regularization parameter (lambda) of 5% was used in both LCMV and DICS analyses. For statistical testing of the source-localizations underlying ERP and TF effects, we used the same cluster-based approach, in this case only clustering over voxels. For plotting purposes, the significant results were interpolated on a template brain which was based on the same anatomy from which the headmodel was created.

## Additional Information

**How to cite this article**: Bögels, S. *et al.* Neural signatures of response planning occur midway through an incoming question in conversation. *Sci. Rep.* **5**, 12881; doi: 10.1038/srep12881 (2015).

## Supplementary Material

Supplementary Information

## Figures and Tables

**Table 1:** Mean, SD, and range (in ms) for the time between the time-locking points and question onset and end.

	**Mean**	**SD**	**Min**	**Max**
onset question - onset critical EARLY (TL1)	1364	591	126	3167
onset question - onset equivalent LATE (TL1)	1379	584	305	3415
onset question - onset critical LATE (TL2)	3071	883	1564	6177
onset question - onset equivalent EARLY (TL2)	3125	917	1580	7180
onset critical EARLY (TL1) - question end	2405	616	1348	4699
onset equivalent LATE (TL1) - question end	2401	725	1438	6129
onset critical LATE (TL2) - question end	655	246	267	1419
onset equivalent EARLY (TL2) - question end	699	233	231	1533

## References

[b1] Sacks H., Schegloff E. A. & Jefferson G. A simplest systematics for the organization of turn-taking for conversation. Language 50, 696–735 (1974).

[b2] Stivers T. *et al.* Universals and cultural variation in turn-taking in conversation. Proceedings of the National Academy of Sciences 106, 10587–10592 (2009). 10.1073/pnas.0903616106 PMC2705608 19553212

[b3] Heldner M. & Edlund J. Pauses, gaps and overlaps in conversations. Journal of Phonetics 38, 555–568 (2010).

[b4] Indefrey P. & Levelt W. J. The spatial and temporal signatures of word production components. Cognition 92, 101–144 (2004). 15037128 10.1016/j.cognition.2002.06.001

[b5] Griffin Z. M. & Bock K. What the eyes say about speaking. Psychological Science 11, 274–279 (2000). 11273384 10.1111/1467-9280.00255 PMC5536117

[b6] Magyari L., Bastiaansen M. C., de Ruiter J. P. & Levinson S. C. Early Anticipation Lies behind the Speed of Response in Conversation. Journal of Cognitive Neuroscience 26, 2530–2539 (2014). 24893743 10.1162/jocn_a_00673

[b7] De Ruiter J. P., Mitterer H. & Enfield N. J. Projecting the end of a speaker’s turn: A cognitive cornerstone of conversation. Language 82, 515–535 (2006).

[b8] Bögels S. & Torreira F. Listeners use intonational phrase boundaries to project turn ends in spoken interaction. Journal of Phonetics 52, 46–57 (2015).

[b9] Torreira F., Bögels S. & Levinson S. C. Breathing for answering: the time course of response planning in conversation. Frontiers in psychology 6 (2015), 10.3389/fpsyg.2015.00284. PMC4357202 25814976

[b10] Kutas M. & Hillyard S. A. Reading senseless sentences: Brain potentials reflect semantic incongruity. Science 207, 203–205 (1980). 7350657 10.1126/science.7350657

[b11] Hagoort P., Brown C. M. & Osterhout L. The neurocognition of syntactic processing in *The neurocognition of language* (eds. Brown C. M. & Hagoort P. ) 273–316 (Oxford University Press, 1999).

[b12] Eulitz C., Hauk O. & Cohen R. Electroencephalographic activity over temporal brain areas during phonological encoding in picture naming. Clinical neurophysiology 111, 2088–2097 (2000). 11068246 10.1016/s1388-2457(00)00441-7

[b13] Habets B., Jansma B. M. & Münte T. F. Neurophysiological correlates of linearization in language production. BMC neuroscience 9 (2008), 10.1186/1471-2202-9-77. PMC2543022 18681961

[b14] Jescheniak J. D., Schriefers H., Garrett M. F. & Friederici A. D. Exploring the activation of semantic and phonological codes during speech planning with event-related brain potentials. Journal of Cognitive Neuroscience 14, 951–964 (2002). 12191461 10.1162/089892902760191162

[b15] Koester D. & Schiller N. O. Morphological priming in overt language production: Electrophysiological evidence from Dutch. NeuroImage 42, 1622–1630 (2008). 18674626 10.1016/j.neuroimage.2008.06.043

[b16] Schmitt B. M., Schiltz K., Zaake W., Kutas M. & Münte T. F. An electrophysiological analysis of the time course of conceptual and syntactic encoding during tacit picture naming. Journal of Cognitive Neuroscience 13, 510–522 (2001). 11388923 10.1162/08989290152001925

[b17] Vos D. M. *et al.* Removal of muscle artifacts from EEG recordings of spoken language production. Neuroinformatics 8, 135–150 (2010). 20480401 10.1007/s12021-010-9071-0

[b18] Strijkers K., Costa A. & Thierry G. Tracking lexical access in speech production: electrophysiological correlates of word frequency and cognate effects. Cerebral cortex 20, 912–928 (2010). 19679542 10.1093/cercor/bhp153

[b19] Pardo J. V., Pardo P. J., Janer K. W. & Raichle M. E. The anterior cingulate cortex mediates processing selection in the Stroop attentional conflict paradigm. Proceedings of the National Academy of Sciences 87, 256–259 (1990). 10.1073/pnas.87.1.256 PMC53241 2296583

[b20] Babiloni C. *et al.* Human movement-related potentials vs desynchronization of EEG alpha rhythm: a high-resolution EEG study. NeuroImage 10, 658–665 (1999). 10600411 10.1006/nimg.1999.0504

[b21] Jensen O. & Mazaheri A. Shaping functional architecture by oscillatory alpha activity: gating by inhibition. Frontiers in Human Neuroscience 4 (2010), 10.3389/fnhum.2010.00186. PMC2990626 21119777

[b22] Adrian E. D. Brain rhythms. Nature 153, 360–362 (1944).

[b23] Fu K.-M. G. *et al.* Attention-dependent suppression of distracter visual input can be cross-modally cued as indexed by anticipatory parieto–occipital alpha-band oscillations. Cognitive Brain Research 12, 145–152 (2001). 11489617 10.1016/s0926-6410(01)00034-9

[b24] Jensen O., Gelfand J., Kounios J. & Lisman J. E. Oscillations in the alpha band (9–12 Hz) increase with memory load during retention in a short-term memory task. Cerebral cortex 12, 877–882 (2002). 12122036 10.1093/cercor/12.8.877

[b25] Yamamoto M. *et al.* Spatially filtered magnetoencephalographic analysis of cortical oscillatory changes in basic brain rhythms during the Japanese ‘Shiritori’Word Generation Task. Neuropsychobiology 53, 215–222 (2006). 16888404 10.1159/000094835

[b26] Jongman S. R., Roelofs A. & Meyer A. S. Sustained attention in language production: An individual differences investigation. The Quarterly Journal of Experimental Psychology 68, 710–730 (2015). 25214187 10.1080/17470218.2014.964736

[b27] Boiteau T. W., Malone P. S., Peters S. A. & Almor A. Interference between conversation and a concurrent visuomotor task. Journal of experimental psychology: General 143, 295–311 (2014). 23421443 10.1037/a0031858 PMC3720820

[b28] Sjerps M. J. & Meyer A. S. Variation in dual-task performance reveals late initiation of speech planning in turn-taking. Cognition 136, 304–324 (2015). 25522192 10.1016/j.cognition.2014.10.008

[b29] Boersma P. & Weenink D. Praat, a system for doing phonetics by computer. Glot International 5, 341–345 (2001).

[b30] Oostenveld R., Fries P., Maris E. & Schoffelen J.-M. FieldTrip: open source software for advanced analysis of MEG, EEG, and invasive electrophysiological data. Computational intelligence and neuroscience 2011 (2011), 10.1155/2011/156869. PMC3021840 21253357

[b31] Grandke T. Interpolation algorithms for discrete Fourier transforms of weighted signals. Instrumentation and Measurement, IEEE Transactions on 32, 350–355 (1983).

[b32] Maris E. & Oostenveld R. Nonparametric statistical testing of EEG-and MEG-data. Journal of neuroscience methods 164, 177–190 (2007). 17517438 10.1016/j.jneumeth.2007.03.024

[b33] Van Berkum J., Hagoort P. & Brown C. Semantic integration in sentences and discourse: Evidence from the N400. Journal of Cognitive Neuroscience 11, 657–671 (1999). 10601747 10.1162/089892999563724

[b34] Oostenveld R., Praamstra P., Stegeman D. & Van Oosterom A. Overlap of attention and movement-related activity in lateralized event-related brain potentials. Clinical neurophysiology 112, 477–484 (2001). 11222970 10.1016/s1388-2457(01)00460-6

[b35] Van Veen B. D. & Buckley K. M. Beamforming: A versatile approach to spatial filtering. ASSP Magazine, IEEE 5, 4–24 (1988).

[b36] Gross J. *et al.* Dynamic imaging of coherent sources: studying neural interactions in the human brain. Proceedings of the National Academy of Sciences 98, 694–699 (2001). 10.1073/pnas.98.2.694 PMC14650 11209067

