# Supplementary material for: Neural signatures of response planning occur midway through an incoming question in conversation
Source: Sci Rep. 2015 Aug 5;5:12881. doi: 10.1038/srep12881 (PMC4525376; doi:10.1038/srep12881)
Supplement: Supplementary Information [file srep12881-s1.pdf]

Supplementary Information

Title: Neural signatures of response planning occur midway through an incoming question in conversation

Authors: Sara Bögels, Lilla Magyari, Stephen C. Levinson

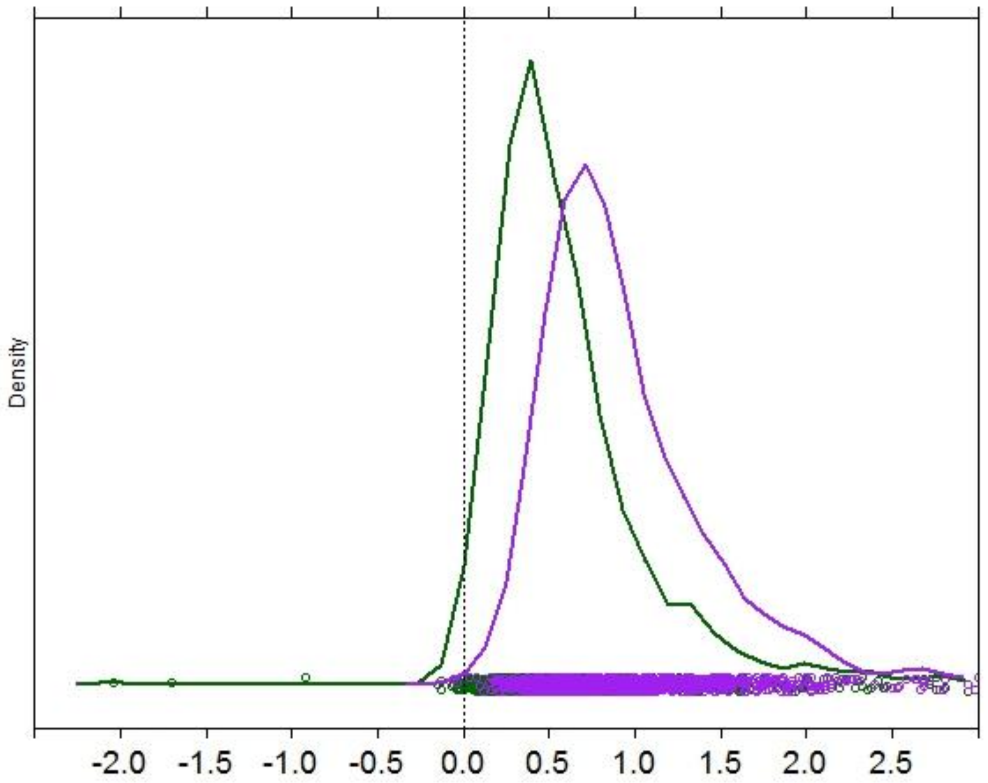

Supplementary Figure 1. Density plot with the distribution of response times relative to question end in seconds. The EARLY condition is indicated with a green line and the LATE condition with a purple line.

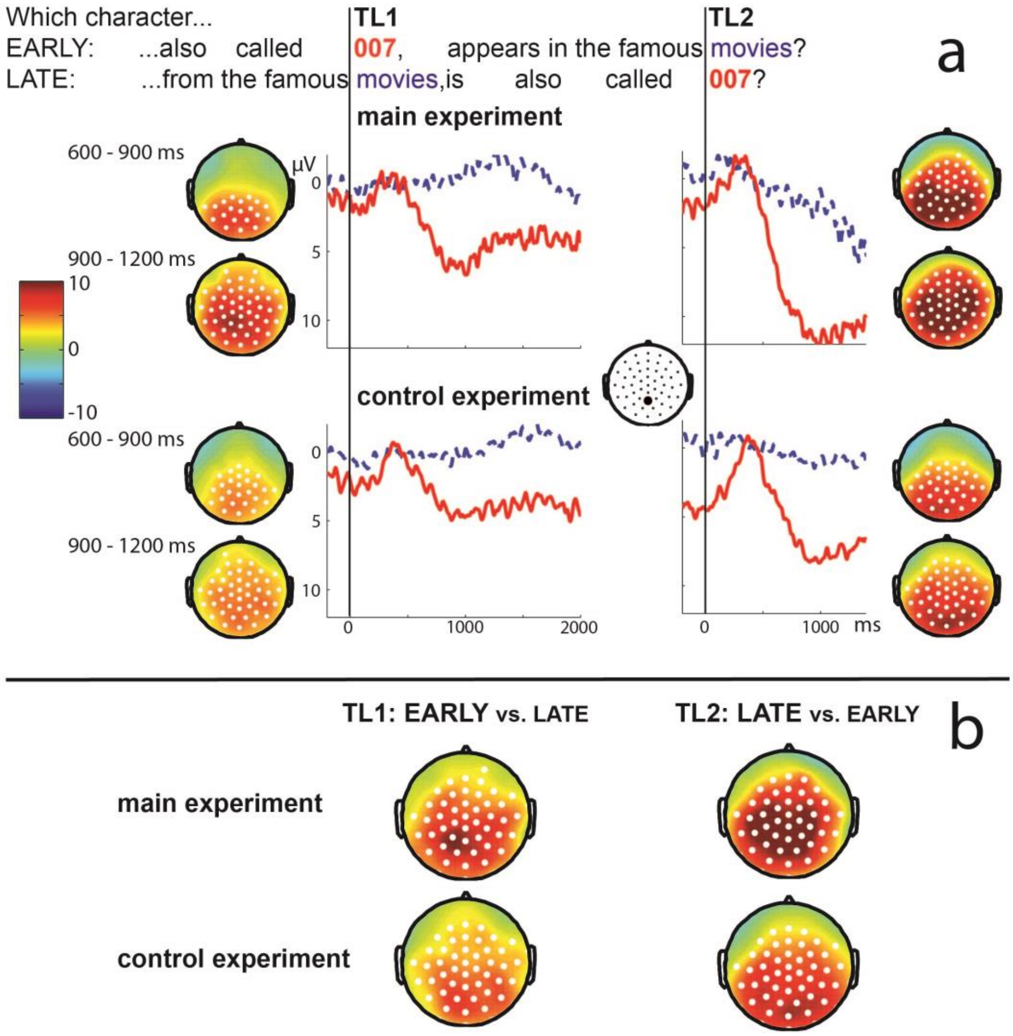

Supplementary Figure 2. Results of the control analyses for the difference in size of the positivity between experiments, taking into account the size of the N400. Panel (a) shows grand average ERPs for a representative electrode (Pz) after baselining to the N400 window (300-500 ms). Critical words are always indicated by red solid lines and equivalent positions by blue dashed lines. Topographical plots are given for two relevant time windows for the positivity (top: 600-900 ms, bottom: 900-1200 ms). Colors indicate T-values. Electrodes that show a significant effect in more than 70% of the time window are highlighted in white. All four comparisons yielded significant positivities starting around 500 ms until the end of the time window (all ps <.001). A comparison between the experiments showed that the

positivities were larger in the main than in the control experiment (TL1: 912-1236 ms; p =.02; TL2: 616-760 ms. p = .01 and 774-1129 m, p = .004). Panel (b) shows topographical plots for a peak-to-peak analysis of the difference between the negative peak (between 350 and 450 ms) and the positive peak (between 950 and 1050 ms). Colors indicate T-values. Electrodes that show a significant effect are highlighted in white. The peak-to-peak difference was larger in the main than in the control experiment (TL1: p =.04; TL2: p = .017).

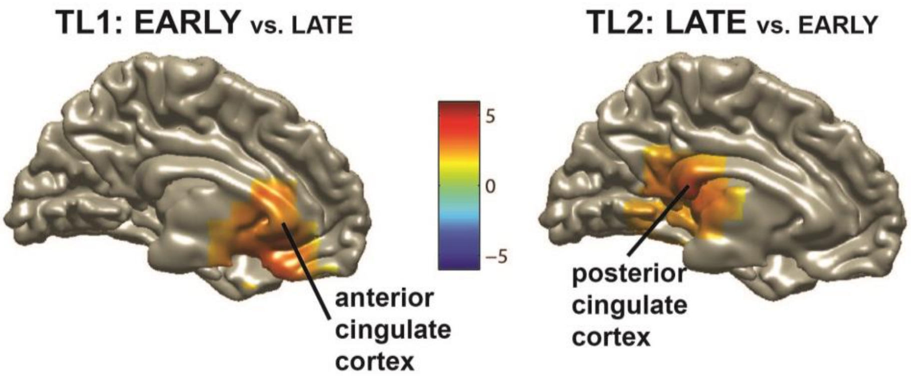

Supplementary Figure 3. Localizations of the positivities in the ERPs (600-1100 ms) of the control experiment. Localizations at TL1 (EARLY vs. LaATE) are shown on the left (one cluster, p = .034) and at TL2 (LATE vs. EARLY) are shown on the right (one cluster, p = .025). Colors indicate T-values.

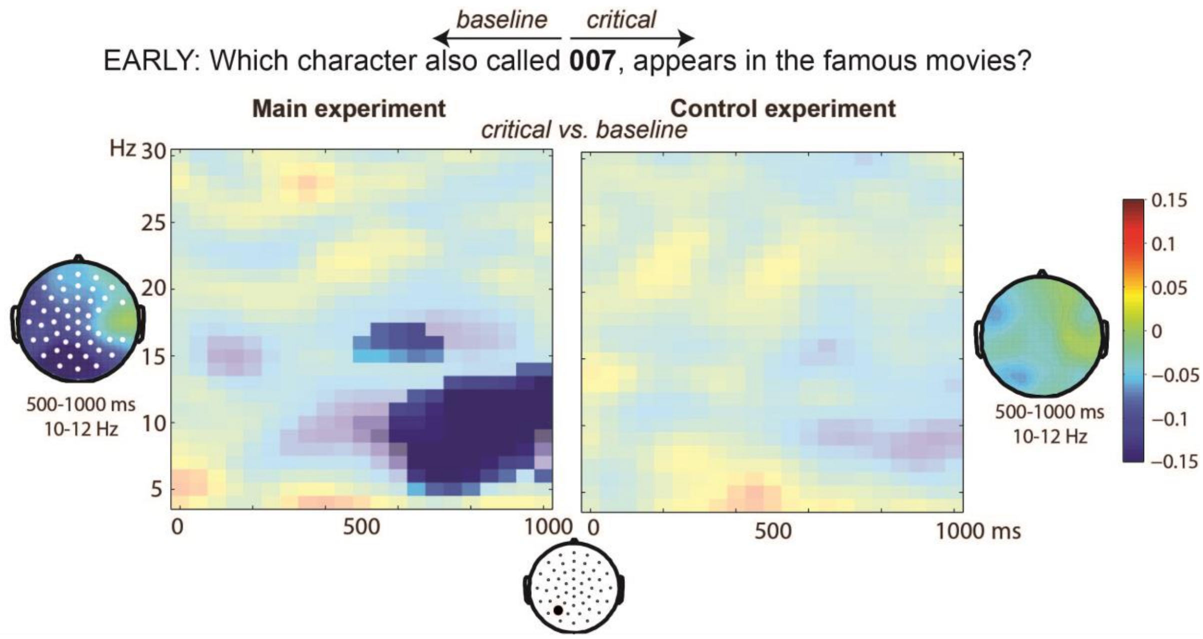

Supplementary Figure 4. Time-frequency results of the critical word versus the baseline period (immediately before the critical word) in the EARLY condition for a representative electrode (left posterior, see middle). The analyses yielded a significant negative cluster for the critical window versus the baseline period in the main experiment ($p < .001$), but not in the control experiment ($p > .1$) and a significant interaction between the two experiments ($p =.002$). Colors in all plots indicate the relative difference between raw power in the relevant conditions. In the time-frequency plots, the relative difference is given in transparent colors with the statistically significant cluster overlaid in opaque colors. Topographical plots are given for 500-1000 ms and the 10-12 Hz range. Electrodes that are significant in the time window are highlighted in white.

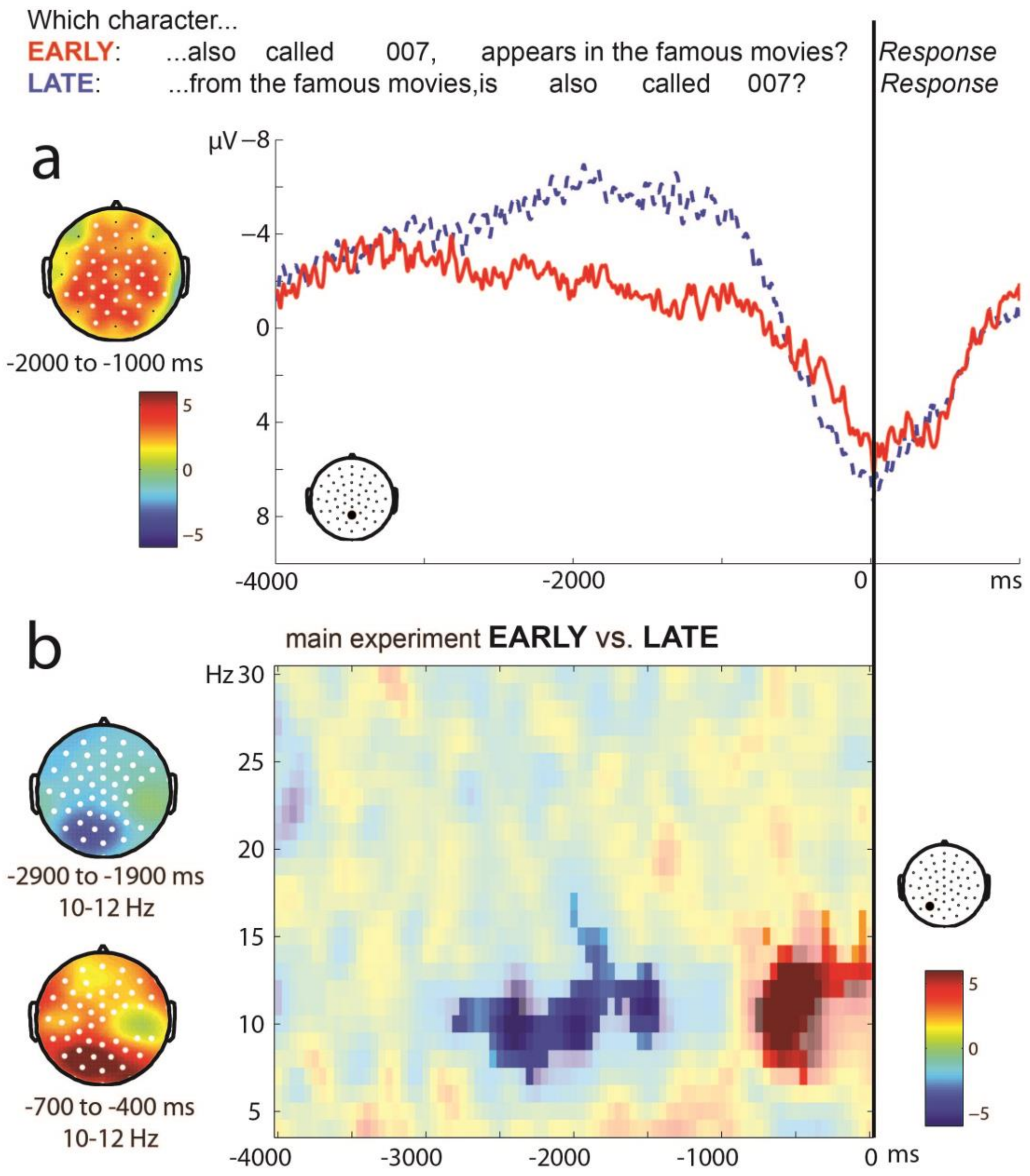

Supplementary Figure 5. Results of the main experiment time-locked to response onset. Panel a shows non-baselined ERPs at a representative electrode (Pz). The early condition is indicated by a red solid line and the late condition by a blue dashed line. Cluster-analyses yielded a positive effect for the early condition between 2926 and 578 ms before the response ($p = .004$). A topographical plot is given for the time window between 2000 and 1000 ms before response. Colors indicate T-values. Electrodes that show a significant effect in more than 70% of the time window are highlighted in white. Panel b shows time-frequency results

time-locked to response onset for a representative left posterior electrode. Colors in all plots indicate the relative difference between raw power in the EARLY relative to the LATE condition. In the time-frequency plots, the relative difference is given in transparent colors with the statistically significant cluster overlaid in opaque colors. Cluster-analyses yielded two clusters around the alpha frequency; one early negative cluster ($p = .006$) reflecting decreased alpha for the EARLY relative to the LATE condition (cf. Figure 3, top left) and one positive cluster ($p = .013$) reflecting decreased alpha for the LATE relative to the EARLY condition (cf. Figure 3, top right). Topographical plots are given for appropriate time windows and for the 10-12 Hz range. Electrodes that participate in a significant cluster in the given time-window are highlighted in white.

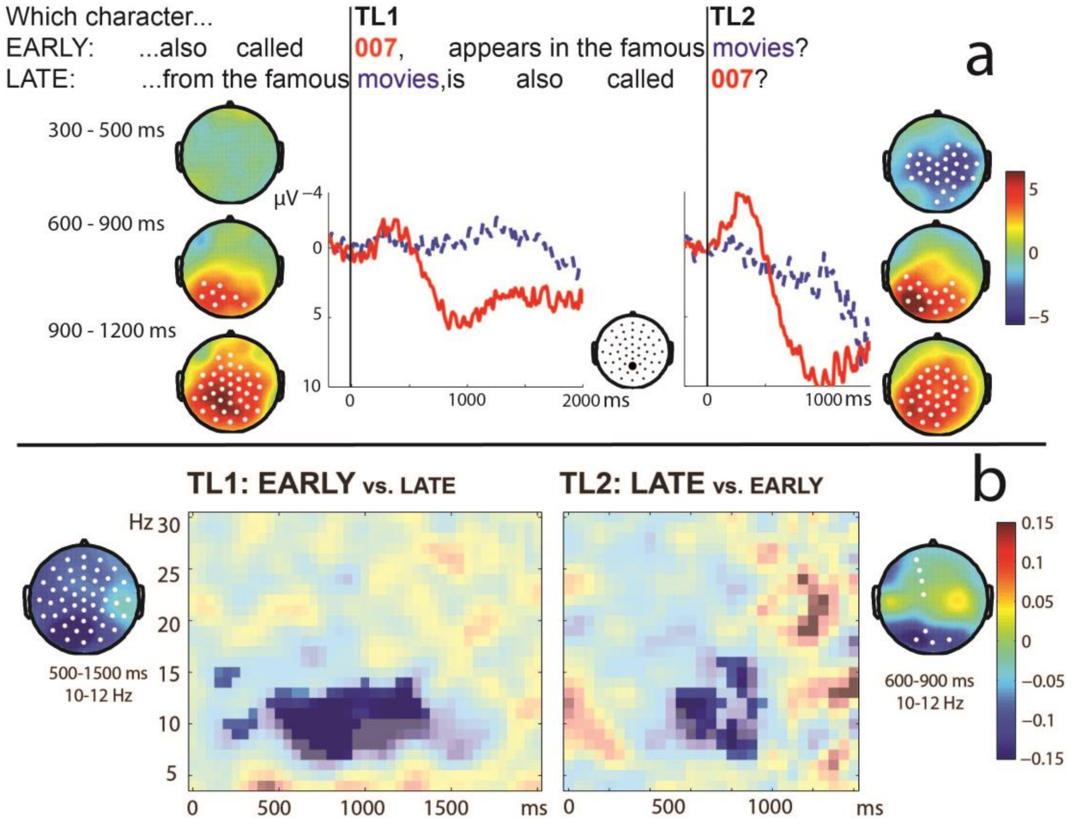

Supplementary Figure 6. Results of the main experiment excluding participants who reported (when probed) that some of the questions might have been recorded. Panel (a) shows grand average ERPs for a representative electrode (Pz). Critical words are always indicated by red solid lines and equivalent positions by blue dashed lines. Cluster analyses at the first time-locking point showed a positive cluster for the critical word in the EARLY condition between 566 and 2000 ms ($p < .001$). Analyses at the second time-locking point showed both a negative cluster between 111 and 718 ms ($p = .003$) and a positive cluster between 520 and 1249 ($p < .001$) for the critical word in the LATE condition. Topographical plots are given for the N400 time-window (top: 300-500 ms) and two time-windows for the positivity (middle: 600-900 ms; bottom: 900-1200 ms). Colors indicate T-values. Electrodes that show a significant effect in more than 70% of the time window are highlighted in white.

Panel (b) shows time-frequency results for a representative electrode (left posterior, see middle). Colors in all plots indicate the relative difference between raw power in the relevant conditions. Cluster-analyses at TL1 yielded a negative cluster for the EARLY relative to the LATE condition around 10 Hz and between about 500 and 1500 ms ($p < .001$). Cluster-analyses at TL2 yielded a negative cluster for LATE relative to the EARLY condition around 10 Hz and between about 600 and 900 ms ($p = .04$). In the time-frequency plots, the relative difference is given in transparent colors with the statistically significant cluster overlaid in opaque colors. Topographical plots are given for appropriate time windows and for the 10-12 Hz range. Electrodes that are significant in the time window are highlighted in white. In sum, the analyses excluding participants who reported that the questions might have been recorded, showed largely the same effects as the main analyses reported in the Results and Discussion (cf. Figures 1 and 3).

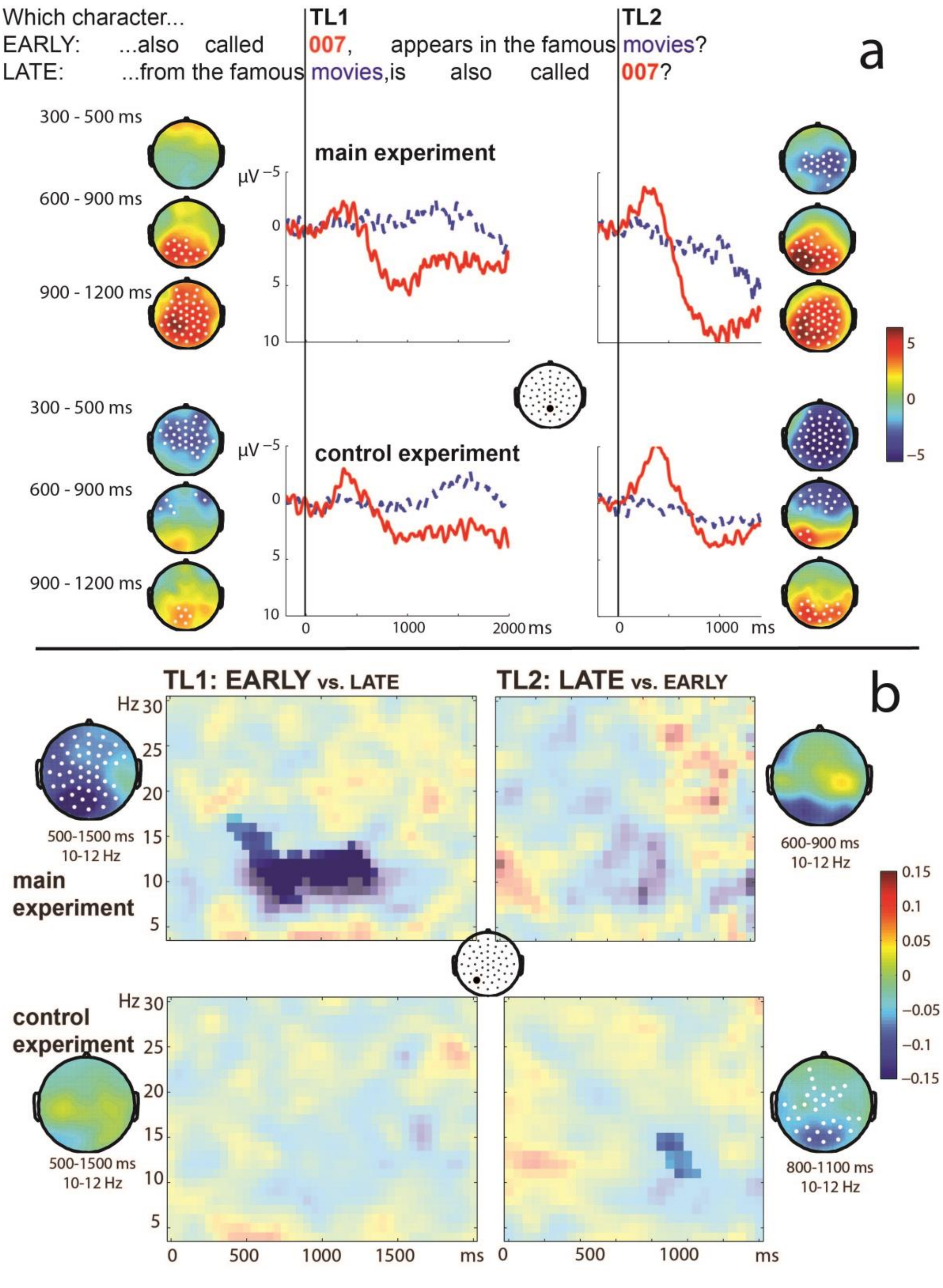

Supplementary Figure 7. Results of the main and control experiment excluding items in which the equivalent position contained a function word. Panel (a) shows grand average ERPs for a representative electrode (Pz). Critical words are always indicated by red solid

lines and equivalent positions by blue dashed lines. Cluster analyses for the main experiment at TL1 showed a positive cluster (EARLY vs. LATE; 592-2000 ms, $p < .001$) and at TL2 showed both a negative cluster (LATE vs. EARLY; 95-518 ms, $p = .005$) and a positive cluster (LATE vs. EARLY; 514-1339, $p < .001$). Cluster analyses for the control experiment at TL1 showed both a negative cluster (EARLY vs. LATE; 250-954 ms, $p = .007$) and a positive cluster (EARLY vs. LATE; 718-2000 ms, = .004) and at TL2 showed both a negative cluster (LATE vs. EARLY; 70-1009 ms, $p < .001$) and a positive cluster (LATE vs. EARLY; 586-1400, $p = .007$). Analyses comparing the two experiments yielded effects at TL1 (negative: 356-574, $p = .02$; positive: 752-1366, $p = .003$) and at TL2 (negative: 354-502, $p = .03$; positive: 510-1125, $p < .001$). Thus, the N400 effects were larger in the control experiment and the positivities were larger in the main experiment. Topographical plots are given for the N400 time-window (top: 300-500 ms) and two time-windows for the positivity (middle: 600-900 ms; bottom: 900-1200 ms). Colors indicate T-values. Electrodes that show a significant effect in more than 70% of the time window are highlighted in white. Panel (b) shows time-frequency results for a representative electrode (left posterior, see middle). Colors in all plots indicate the relative difference between raw power in the relevant conditions. Cluster-analyses for the main experiment at TL1 yielded a negative cluster for the critical word in the EARLY condition around 10 Hz and between about 500 and 1500 ms ($p = .003$), while analyses at TL2 did not yield any significant clusters ($p > .3$). Cluster-analyses for the control experiment at TL1 yielded no significant clusters ($p > .3$), but for TL2 yielded a negative cluster for the critical word in the LATE condition around 10 Hz and between about 800 and 1100 ms ($p = .048$). Analyses comparing the two experiments yielded a negative cluster at TL1 ($p < .001$) but no differences between experiments at TL2 ($p > .3$). Thus, the reduction in alpha was larger for the main than the control experiment at TL1. In the time-frequency plots, the relative difference is given in transparent colors with the statistically

significant cluster overlaid in opaque colors. Topographical plots are given for appropriate time windows and for the 10-12 Hz range. Electrodes that are significant in the time window are highlighted in white. In sum, the analyses excluding function words showed largely the same effects as the main analyses reported in the Results and Discussion (cf. Figures 1 and 3).
